# Serotonin Pathway in Cancer

**DOI:** 10.3390/ijms22031268

**Published:** 2021-01-28

**Authors:** Pragathi Balakrishna, Sagila George, Hassan Hatoum, Sarbajit Mukherjee

**Affiliations:** 1 Stephenson Cancer Center, University of Oklahoma Health Sciences Center, Oklahoma City, OK 73104, USA; Pragathi-Balakrishna@ouhsc.edu (P.B.); Sagila-George@ouhsc.edu (S.G.); 2 Roswell Park Comprehensive Cancer Center, Buffalo, NY 14263, USA

**Keywords:** serotonin, 5-HT, 5-HT receptors, cancer, carcinogenesis

## Abstract

Serotonin (5-hydroxytryptamine, 5-HT) is a biogenic monoamine produced from the essential amino acid tryptophan. Serotonin’s role as a neurotransmitter in the central nervous system and a motility mediator in the gastrointestinal tract has been well defined, and its function in tumorigenesis in various cancers (gliomas, carcinoids, and carcinomas) is being studied. Many studies have shown a potential stimulatory effect of serotonin on cancer cell proliferation, invasion, dissemination, and tumor angiogenesis. Although the underlying mechanism is complex, it is proposed that serotonin levels in the tumor and its interaction with specific receptor subtypes are associated with disease progression. This review article describes serotonin’s role in cancer pathogenesis and the utility of the serotonin pathway as a potential therapeutic target in cancer treatment. Octreotide, an inhibitor of serotonin release, is used in well-differentiated neuroendocrine cancers, and the tryptophan hydroxylase (TPH) inhibitor, telotristat, is currently being investigated in clinical trials to treat patients with metastatic neuroendocrine tumors and advanced cholangiocarcinoma. Several in vitro studies have shown the anticancer effect of 5-HT receptor antagonists in various cancers such as prostate cancer, breast cancer, urinary bladder, colorectal cancer, carcinoid, and small-cell lung cancer. More in vivo studies are needed to assess serotonin’s role in cancer and its potential use as an anticancer therapeutic target. Serotonin is also being evaluated for its immunoregulatory properties, and studies have shown its potential anti-inflammatory effect. Therefore, it would be of interest to explore the combination of serotonin antagonists with immunotherapy in the future.

## 1. Introduction

Serotonin or 5-hydroxytryptamine (5-HT) was isolated and characterized in 1948 by Maurice Rapport and Irvine Page. The name serotonin was derived from the Latin word serum and the Greek word tonic [1]. 5-HT is a monoamine produced from the essential amino acid tryptophan. It is synthesized in two steps catalyzed by the enzymes tryptophan hydroxylase (TPH) and dopa decarboxylase (DDC), TPH being the rate-limiting enzyme. The mammalian brain and the peripheral sites synthesize serotonin separately as it cannot cross the blood–brain barrier. This is facilitated by the rate-limiting enzyme TPH, which exists in two forms, TPH-1 and TPH-2. TPH-1 is usually found in the pineal body and the digestive tract, and TPH-2 is selectively expressed in the brain [2]. Outside of the central nervous system (CNS), serotonin synthesis is mostly restricted to intestinal enterochromaffin cells and, to a lesser extent, platelets. Platelets have very little ability to synthesize 5-HT, but they represent a major storage site for serotonin. About 90–95% of the body’s serotonin is located in the periphery, mostly intracellularly in platelets, and less than 1% of total body serotonin circulates in its free form in the blood. Within the CNS, serotonin is synthesized and stored in presynaptic neurons [1].

The functions of serotonin are broad, diverse, and sometimes opposing. These effects of serotonin are mediated through several specific 5-HT receptors. These receptors are widely expressed throughout the body, and to date, seven receptor classes (5-HT1-7) have been identified. Most of the receptor classes are heterogeneous and are further subclassified. Overall, 13 receptor subtypes have been identified [3]. Six of these 5-HT receptor classes are G-protein-coupled receptors, but the 5-HT3 receptor is unique as it involves a ligand-gated Na+/K+ ion channel [1]. The function exerted by a particular receptor is determined by the receptor characteristics and the signaling pathway coupled to it. In the CNS, the serotonergic neurons’ cell bodies are located in the brain stem’s nine raphe nuclei. These neurons give rise to broad projections to the forebrain, hindbrain, and spinal cord [4].

The most clinically relevant function of serotonin in the CNS is its role in psychological disorders such as depression, mania, and anxiety disorders. Therefore, many pharmaceutical drugs, including antidepressants and antipsychotics, have been developed to aim at the serotonergic system [5]. Other known functions of serotonin include the regulation of intestinal motility and emesis, vasoconstriction, amplification of platelet aggregation, and wound healing [6]. Table 1 summarizes the different 5-HT receptors based on their location and function.

More recently, serotonin has demonstrated carcinogenic properties. This has sparked further research into its potential role at different stages of tumor progression and the utility of 5-HT receptor antagonists, serotonin synthesis inhibitors, and serotonin transporter (SERT) to prevent cancer growth [7].

The present review aims to redefine serotonin’s role in cancer pathogenesis and the serotonin pathway’s utility as a potential therapeutic target in cancer treatment. In addition, we shall shed light on the ongoing clinical trials targeting this pathway.

## 2. Involvement of Serotonin in Carcinogenesis

Different studies have demonstrated serotonin’s growth-stimulatory effect on several types of cancers and carcinoids [7]. This review highlights serotonin’s role in prostate cancer, breast cancer, small-cell lung cancer, colorectal cancer, cholangiocarcinoma, hepatocellular carcinoma, glioma, and carcinoid tumors.

### 2.1. Prostate Cancer

Prostate cancer (PC) is the most frequent cancer in males, and hormone-refractory metastatic prostate cancer represents this progressive malignancy’s end stage. Neuroendocrine cells (NE) are epithelial cell types in the normal prostate gland and basal and exocrine secretory cells. NE cells are also present in most prostate cancers, including metastatic PC. These cells produce and secrete serotonin, along with other peptide hormones [7]. It has been proposed that NE cells might facilitate tumor recurrence by assisting proliferation of neighboring non-NE cells in PC, and some studies have suggested that the number of NE cells correlate with stage and Gleason score, thereby predicting poor prognosis and shortened patient survival [8,9]. Serotonin has been detected in benign prostate tissue, PC, and in metastases. Its action on PC is mediated through different receptor subtypes at different tumor stagesStrong expression of 5-HT1A and 5-HT1B receptors has been seen in aggressive PC with a high Gleason score and metastatic PC [10]. 5-HT4 receptors have been predominantly found in high-grade tumors. On the other hand, 5-HT2B receptor expression has been associated with pathologically altered prostate tissue, suggesting that this is involved in the early stages of PC [11]. These studies support the hypothesis that NE cells and serotonin secretion may promote prostate cell growth in an androgen-depleted environment via serotonin-induced growth factor release. An in vitro study using an androgen-independent cell line showed that serotonin caused a dose-dependent stimulatory effect on cell proliferation [12]. Another in vitro study observed that serotonin activates Mitogen activated protein (MAP) kinase and PI3K/Akt signaling pathways involved in PC cell migration. It also noted that cell lines pretreated with a selective 5-HT1A receptor antagonist had a reduced upregulation of signal transduction pathways and reduced cell proliferation and migration [13]. The above studies show that hormone-independent cell lines are sensitive to 5-HT receptor antagonists, with the 5-HT1B antagonist being the more likely therapeutic option for further research [12]. However, another study using androgen-responding cell lines does not support the hypothesis of 5-HT1A and 1B receptors in mediating PC cell growth at serotonin’s physiological concentration [14]. These inconsistent results are probably due to 5-HT receptor subtypes’ involvement with a lower affinity for serotonin [7].

### 2.2. Breast Cancer

Breast cancer prognosis markers have been limited so far. This opens up an exciting avenue to search for prognostic as well as therapeutic parameters. Serotonin’s role in mammary gland development and as a regulator for epithelial homeostasis in normal breast tissue during pregnancy, lactation, and involution has been well described. Dysregulation of epithelial homeostasis has been associated with breast cancer initiation and progression [7]. Serotonin promotes the growth of neoplastic breast cells partly through 5-HT2A receptors. This was seen in the human breast adenocarcinoma cell line MCF-7, where serotonin and the selective 5-HT2A receptor agonist stimulated cell growth in a concentration-dependent manner [15]. It has also been demonstrated that TPH1 expression is increased during tumor progression, which corresponds to increased serotonin synthesis [16]. Tissue microarray in 102 breast cancer patients was performed to study the expression of various 5-HT receptors. High expression of 5-HT1A was seen in the plasma membrane of breast cancer cells but was also observed mainly in the cytoplasm of nonmalignant cells. 5-HT1B and 2B expression was variable and was observed in the cytoplasm of both malignant and nonmalignant cells. A substantial correlation between 5-HT2B and estrogen-α and between 5-HT4 and estrogen-α and progesterone receptors was also identified. Nevertheless, no correlation between receptor subtype and grade of tumor was found. This study’s results were highly variable, and further studies are needed to establish a strong correlation between 5-HT receptor subtypes and tumor progression and establish the utility of serotonin as a prognostic marker [17].

A recent study investigated changes in mRNA expression of 5HTR2A and 5HTR3A receptors in the breast tumor tissue compared to their marginal zone. This study showed an increased expression of the above receptor genes in breast tumor tissue than marginal tissue, indicating that the mitogenic nature of 5HT receptors leads to increased proliferation of cancer cells [18].

Depression is a common symptom experienced by a majority of patients with breast cancer. This is generally treated with antidepressant (AD) medications such as selective serotonin reuptake inhibitors (SSRI) or tricyclic antidepressants (TCA). An animal study noted that TCA and SSRI might promote tumor growth and increase breast cancer risk [19], but this was not confirmed by human or in vitro studies. A meta-analysis looking at the association between AD and the risk of breast cancer was conducted, and the overall risk of breast cancer did not increase among AD users [20]. However, it has been suggested that SSRIs may inhibit cell proliferation. One study showed that SSRI fluoxetine might have an anticarcinogenic effect and enhance chemosensitivity [21].

### 2.3. Small-Cell Lung Cancer (SCLC)

SCLC is a very aggressive epithelial tumor with early metastasis and is associated with tobacco use. It has been shown that SCLC shows properties of NE differentiation [7]. Nicotine stimulates the proliferation of SCLC cells, and being a strong secretagogue, it also stimulates the release of serotonin from these cells. Serotonin’s involvement in SCLC proliferation was observed in an in vitro study wherein serotonin’s addition to SCLC cell lines induced cell proliferation in a dose-dependent manner, and a 5-HT1 receptor antagonist blocked this effect. These data suggest that a serotonergic pathway may be involved in the proliferation of SCLC [22]. Subsequent work has shown the involvement of 5-HT1D and 5-HT1A receptor subtypes in serotonin’s mitogenic effect [23]. The possibility of inhibiting the serotonergic autocrine loop as a therapeutic modality for SCLC was evaluated in the 1990s. However, serotonin’s mitogenic effect on human SCLC cells was found to be complicated, with possible interactions between 5-HT1A and 5-HT1D receptors [23]. Nevertheless, the importance of serotonin on SCLC progression cannot be overlooked, and it offers a promising therapeutic target for further research.

### 2.4. Colorectal Cancer (CRC)

In murine models, it was found that elevated serotonin levels activated lymphocytes leading to cytokine release, which mimicked human inflammatory bowel disease. This suggests that a serotonin-mediated pro-inflammatory microenvironment may be responsible for colorectal tumorigenesis [24]. Another animal study demonstrated that an intraperitoneal injection of serotonin increased the mitotic rate in descending colon adenocarcinoma cells in rats, and the TPH inhibitor was shown to decrease tumor cell mitotic rate [7]. However, more recently, mouse models defective in 5-HT synthesis were used to investigate the early mutagenic events associated with CRC. Surprisingly, they reported a novel protective role of serotonin that promoted DNA repair in the early stages of colorectal carcinogenesis [25]. These conflicting results denote that further research is needed to elucidate the exact mechanism of action of serotonin in CRC.

It has been suggested that serotonergic antagonists may prevent cancer cell growth following antineoplastic therapy, and SSRIs may inhibit tumor growth through a direct cytotoxic effect. Daily SSRI has been suggested as a prophylactic agent for patients at high risk of developing CRC [26]. It has also been suggested that the 5-HT1B receptor antagonist increases apoptosis of CRC cells and can be considered a potential target for therapy [27].

### 2.5. Cholangiocarcinoma

Real-time PCR analysis showed an increased expression of TPH1 and decreased Monoamine Oxidase A (MAO-A) expression in human cholangiocarcinoma cell lines compared with nonmalignant cell lines. This was also confirmed by immunohistochemistry (IHC) analysis of human liver biopsies of cholangiocarcinoma. Hence, an increased synthesis of serotonin from cholangiocarcinoma was observed in vitro and in vivo. Human CC cell lines were also found to express all 5-HT receptor subtypes. Specific inhibition of 5-HT1A, 2A, 2B, 4, and 6 receptors was associated with antiproliferative effects.

Furthermore, inhibition of serotonin synthesis blocked the growth of CC cell lines [28]. This offers a promising target for future therapies in cholangiocarcinoma. Telotristat ethyl (TE), a TPH inhibitor currently FDA-approved for carcinoid syndrome diarrhea, is being studied in an ongoing phase II study in combination with first-line chemotherapy in patients with advanced cholangiocarcinoma (NCT03790111, Clinicaltrials.gov). The study has completed accrual. Results are being awaited.

### 2.6. Hepatocellular Carcinoma (HCC)

Serotonin initiates liver regeneration after partial hepatectomy and promotes tissue repair after ischemic injury via a pathway dependent on vascular endothelial growth. However, there is increasing evidence that serotonin is involved in many pathological conditions of the liver [29]. It was found to promote cell survival and proliferation of human HCC cell lines Huh7 and HepG2 in a dose-dependent manner [7]. These effects of serotonin are mediated through 5-HT receptors. Among 176 patients with HCC, 5-HT1B and 5-HT2B receptors were expressed in 32% and 35%, respectively. Both these receptors were associated with an increased Ki67 and correlated with the size of the tumor. Their antagonist showed potent cytotoxic effects on HepG2 cell lines [30], thereby suggesting that 5-HT receptors may represent a new therapeutic target for patients with HCC. Proposals for targeting this pathway combined with the standard treatment of HCC, whether in the first-line or second-line, are a potential area of exploration.

### 2.7. Glioma

Glioblastoma (GBM) is the most common form of malignant glioma in adults. It has a poor prognosis with an estimated OS time of 16–18 months. Therapeutic options for GBM are limited. Chemotherapy is less efficacious, and there is a dire need for newer medications. Recent studies have demonstrated that antidepressants such as SSRIs increase intracellular calcium (Ca2+) levels in astrocytes, thereby inducing mitochondrial damage and astrocyte apoptosis. This process may be involved in the pathogenesis of neurodegenerative disorders and cytotoxic effects in certain cancers [31]. In the past few years, in vivo studies have been done to study the potential benefit of SSRIs in treating GBM. Fluoxetine was studied in glioma cell lines and was found to interact with AMPA receptors on cells, thus inducing Ca2+ influx and triggering cell death. AMPA receptors are excessively expressed in glioma tissue, suggesting that fluoxetine suppressed the growth of GBM in the brains of Nu/Nu mice [32]. Similarly, another animal study suggested that escitalopram inhibits the proliferation of xenografted GBM in BALB/c nude mice [33]. These findings imply that SSRIs may have a potential role in the treatment of GBM.

Recent research efforts in GBM are aimed at newer treatment modalities. So far, immunotherapy has not shown a significant OS benefit in GBM, which could be attributed to increased stress levels in patients with brain tumors. It has been well established that psychological distress leading to enhanced adrenergic signaling, inflammation, and immune dysregulation can facilitate tumor growth [34]. A meta-analysis reported that glioma patients with depression had significantly worsened OS [35]. This led to a theory that treating psychological distress with SSRIs may improve survival in GBM patients, although a retrospective review of 497 patients failed to find an association between SSRI use and OS in patients with GBM [36].

Along with the direct cytotoxic effects of SSRIs on astrocytes seen in animal models, it is worthwhile to study further the association between stress-signaling pathways and SSRIs. If immune dysregulation caused by stress can be reversed by SSRIs, then they may play a role as an adjunct therapy to increase the response to immunotherapy.

### 2.8. Carcinoid Tumors

Increased serotonin secretion is seen in many NE tumors (NET). Serotonin that reaches the systemic circulation by bypassing hepatic inactivation is responsible for producing carcinoid syndrome. This syndrome is typically associated with NE of midgut and rarely seen with NE of hindgut and foregut. In vitro studies evaluating serotonin’s proliferative cell role in multiple NE cell lines suggest that serotonin’s proliferative effect is mediated via 5-HT1A and 1B receptors in the pancreatic tumor 5-HT2 receptors in bronchopulmonary NET and small intestinal NET [37]. Somatostatin analogs (SSAs) were first introduced for symptomatic management of carcinoid syndrome caused by NET. Landmark findings of the PROMID trial demonstrated the role of octreotide as an antiproliferative agent and, in some cases, even to reduce tumor burden in metastatic carcinoids [38]. The more recent CLARINET phase III trial showed that lanreotide was associated with a significantly prolonged progression-free survival in patients with metastatic enteropancreatic NET [39]. Based on these data, SSA is a cornerstone of the first-line treatment for NETs either as a single agent or in combination therapy. Telotristat ethyl (TE), a tryptophan hydroxylase (TPH) inhibitor, has also been shown to significantly decrease carcinoid syndrome diarrhea in patients with refractory carcinoid [40]. Ongoing studies of TE in combination with SSAs (TELEFIRST study) in the first-line setting are likely to provide more data on its antiproliferative effect in NETs.

## 3. Role of Serotonin in Tumor Vasculature and Angiogenesis

Serotonin at physiological doses is known to function as a potent angiokine. It has been postulated that the thrombotic environment of tumors induces platelet aggregation, leading to serotonin release, hence promoting angiogenesis and tumor growth [41]. A study involving a mouse model of tumor allograft showed that serotonin is a regulator of angiogenesis by suppressing MMP-12 expression in tumor-infiltrating macrophages, thus inhibiting angiostatin, an angiogenesis suppressor in solid tumors [42].

Serotonin’s effect on tumor vasculature is a complex process and depends on its interaction with various 5-HT receptors. Several studies have focused on serotonin’s action on the vascular tone of the arterioles feeding the tumor. This is dependent on the dominant receptor in the particular tumor and the concentration of serotonin present. For example, serotonin mediated vasoconstriction is caused by its interaction with 5-HT1B and 2A receptors on vascular smooth muscles, while serotonin mediated vasodilation is due to its interaction with the 5-HT2B receptor present on endothelial cells [43]. In a study of SCLC grafted in nude mice, it was observed that serotonin exerted opposing dose-dependent effects on tumor growth. It exerted a mitogenic effect at higher doses, whereas, at lower doses, it reduced tumor growth via its effect on tumor vasculature [44].

This opens up the possibility of using these receptors as potential targets to inhibit tumor growth. An in vitro study on HUVEC cells showed that 5-HT2B receptors’ inhibition suppressed tumor angiogenesis and thereby reduced implanted lung cancer growth [45]. Another study suggested that the 5-HT4 receptor agonist has anti-angiogenic activity [46]. Further research is needed to verify its role in cancer treatment.

## 4. Role of Serotonin in Immune Dysregulation

Serotonin has complex interactions and influences on immune cells. It plays an essential role in inflammation and immunomodulatory diseases such as gut inflammation, allergic asthma, rheumatoid arthritis, and neurodegenerative diseases.

Immune cells express 5-HT receptors, SERT, and TPH. It has been reported in multiple studies that serotonin exerted complex effects on cytokine release from macrophages and monocytes [47] and hence is a crucial factor in controlling the immune microenvironment. Table 2 [47] summarizes the interaction between serotonin and components of the immune system.

Many studies in the past decade have increased our understanding of the link between inflammation and cancer development. It is now understood that downstream signaling from inflammatory pathways controls carcinogenesis [48]. Therefore, serotonin-induced immune response can be another mechanism of cancer progression.

## 5. Serotonin Pathway as a Potential Therapeutic Target

Apart from the already well-established serotonin functions as a neurotransmitter and its role in several psychiatric and neurological disorders, the serotonergic pathway has been implicated in tumorigenesis more recently. Several in vitro and in vivo studies have demonstrated the role of serotonin and 5-HT receptor subtypes in cell proliferation, angiogenesis, invasion, migration, and metastasis. Genetic models of multiple cancer cells, including lung cancer cells, melanoma cells, and CRC, have all demonstrated that serotonin levels in tumors played a crucial role in tumor growth.

Although serotonin acts as an oncogene, its effect on tumor growth is unclear and complicated. This may be explained by the fact that its actions are mediated through 5-HT receptors whose expression may be tissue-specific. Another hypothesis is that serotonin’s mitogenic effect is dose-dependent, where higher doses promote cell proliferation and lower doses cause vasoconstriction on tumor vessels, leading to inhibition of tumor growth. By contrast, several genetic studies have shown a decreased 5-hydroxytryptamine receptor 1B gene (HTR1B) in the lung, renal, osteosarcoma, and non-Hodgkin’s lymphoma, suggesting that serotonin may behave as a tumor suppressor when it interacts with 5-HTB receptor subtypes [49].

Some data suggest that 5-HT receptor expression is not only tissue-specific but also becomes dysregulated in human cancers. For example, in human cholangiocarcinoma, cell lines 5-HT 1B, 1F, 2B, 3C, and 7 are downregulated, whereas all the other subtypes are up-regulated [28]. 5-HT1B and 2B receptor subtypes are significantly overexpressed in liver tumor cells compared to normal liver cells [30]. Therefore, thorough analysis and a better understanding of 5-HT receptor subtypes in various cancers can help to develop effective targeted therapies.

5-HT receptors, SERT, and serotonin synthesis pathways have been studied as potential pharmacotherapy targets. Numerous serotonin-targeting drugs such as SSRIs are available for treating CNS disorders, and now their utility as anticancer agents is being evaluated. Although SSRIs increase serotonin levels in synaptic cleft and plasma, surprisingly, they do not contribute to tumorigenesis. Instead, certain SSRIs have demonstrated cytotoxic effects in vitro but at higher concentrations. Thus, their use as anticancer agents may be limited since concentrations obtained in patients treated with antidepressant doses of SSRIs are much lower.

Other agents targeting the serotonin pathway include SSAs. Octreotide and lanreotide are currently approved for treating carcinoid syndrome associated with NET, and their antiproliferative effect in metastatic NET is also well established. More recently, TE, a TPH inhibitor, is being evaluated in metastatic NET and other cancers such as cholangiocarcinoma.

Apart from serotonin’s above functions, more recently, there is increasing evidence that serotonin interacts with the peripheral immune system, particularly with T cells. Although the exact mechanism of serotonin signaling in the immune system is unclear, studies do suggest that 5-HT has an immune-stimulatory effect, and the 5-HT1A receptor antagonists decreased T-cell proliferation and cytokine production in vitro. It was also observed that SSRI fluoxetine may exhibit an immunosuppressive role in T cells [50]. To our knowledge, the immunomodulatory effect of serotonin on human cancers is mostly unknown. In the era of immunotherapy (IO), it would be interesting to see if a 5-HT targeting agent can potentiate IO’s effect in various cancers.

## 6. Conclusions

In conclusion, initial in vivo and in vitro studies targeting the serotonin pathway have shown some promising results. It would be greatly beneficial to develop 5-HT receptor antagonists and explore TPH inhibitors’ and SSRIs’ potential roles as targeted therapies to treat and prevent solid malignancies.

## Figures and Tables

**Table 1:** Serotonin receptor characteristics.

Receptor Type	Signal Transduction Pathway	Subtype	Location	Response
5-HT1	G_1_/G_0_-Adenylyl cyclase	1A	Mainly CNS	Neuronal hyperpolarization
	G_1_/G_0_-Adenylyl cyclase	1B	CNS and peripheral nerves	Inhibits neurotransmitter release
	G_1_/G_0_-Adenylyl cyclase	1D	Mainly CNS	Inhibits neurotransmitter release
	G_1_/G_0_-Adenylyl cyclase	1E	CNS	Inhibits adenylyl cyclase
	G_1_/G_0_-Adenylyl cyclase	1F	Mainly CNS	Inhibits adenylyl cyclase
5-HT2	G_q_/G_11_-Phospholipase C	2A	Vascular smooth muscles, platelets, lung, CNS, GI tract	Vasoconstriction, platelet aggregation, broncho-constriction
	G_q_/G_11_-Phospholipase C	2B	Mainly peripheral	Rat stomach muscle contraction
	G_q_/G_11_-Phospholipase C	2C	CNS (concentrated in choroid plexus)	Increases turnover of phosphoinositide
5-HT3	Ligand-gated cation channel	-	Peripheral and central neurons	Depolarization
5-HT4	G_s_-Adenylyl cyclase	-	GI tract, CNS, heart, urinary bladder	Acetylcholine release in gut, tachycardia, release cAMP in CNS neurons
5-HT5	G_1_/G_0_- Adenylyl cyclase	5A	CNS	Unknown
		5B	Absent in human	
5-HT6	G_s_ -Adenylyl cyclase	-	CNS	Activates adenylyl cyclase
5-HT7	G_s_ -Adenylyl cyclase	-	CNS	Activates adenylyl cyclase

**Table 2:** Functions of serotonin in inflammation and immunity.

Immune Cell	5-HT Receptors	Response
Monocytes and Macrophages Dendritic cells	1A, 1E, 2A, 3A, 4, 7 1B, 1E, 2A, 2B, 4, 7	Releases IL-6, 1β, 8/CXCL8, IFN-γ induced phagocytosis, T-cell stimulation, inhibits release of TNF- α, inhibits NK cell suppression.
Neutrophils	1A, 1B, 2	Inhibits tumor cell phagocytosis and oxidative burst.
T cells	1A, 1B, 2A, 2C, 3A, 7	Releases IL-2, 16 and IFN- γ, T-cell proliferation.
B cells	1A, 2A, 3, 7	
Endothelial cells		T-cell chemoattractant release, eNOS expression, inhibits leukocyte diapedesis
Vascular smooth muscle cells		IL-6 synthesis, inhibits TNF-α induced expression of ICAM-1, VCAM-1, NO, NFκB.
Microglia	2B, 5A, 7	Promotes injury-induced microglial motility, brain maturation
